# Assessing Google Flu Trends Performance in the United States during the 2009 Influenza Virus A (H1N1) Pandemic

**DOI:** 10.1371/journal.pone.0023610

**Published:** 2011-08-19

**Authors:** Samantha Cook, Corrie Conrad, Ashley L. Fowlkes, Matthew H. Mohebbi

**Affiliations:** 1 Google, Inc., New York, New York, United States of America; 2 Google, Inc., London, United Kingdom; 3 Influenza Division, Centers for Disease Control and Prevention, Atlanta, Georgia, United States of America; University of Hong Kong, Hong Kong

## Abstract

**Background:**

Google Flu Trends (GFT) uses anonymized, aggregated internet search activity to provide near-real time estimates of influenza activity. GFT estimates have shown a strong correlation with official influenza surveillance data. The 2009 influenza virus A (H1N1) pandemic [pH1N1] provided the first opportunity to evaluate GFT during a non-seasonal influenza outbreak. In September 2009, an updated United States GFT model was developed using data from the beginning of pH1N1.

**Methodology/Principal Findings:**

We evaluated the accuracy of each U.S. GFT model by comparing weekly estimates of ILI (influenza-like illness) activity with the U.S. Outpatient Influenza-like Illness Surveillance Network (ILINet). For each GFT model we calculated the correlation and RMSE (root mean square error) between model estimates and ILINet for four time periods: pre-H1N1, Summer H1N1, Winter H1N1, and H1N1 overall (Mar 2009–Dec 2009). We also compared the number of queries, query volume, and types of queries (e.g., influenza symptoms, influenza complications) in each model. Both models' estimates were highly correlated with ILINet pre-H1N1 and over the entire surveillance period, although the original model underestimated the magnitude of ILI activity during pH1N1. The updated model was more correlated with ILINet than the original model during Summer H1N1 (r = 0.95 and 0.29, respectively). The updated model included more search query terms than the original model, with more queries directly related to influenza infection, whereas the original model contained more queries related to influenza complications.

**Conclusions:**

Internet search behavior changed during pH1N1, particularly in the categories “influenza complications” and “term for influenza.” The complications associated with pH1N1, the fact that pH1N1 began in the summer rather than winter, and changes in health-seeking behavior each may have played a part. Both GFT models performed well prior to and during pH1N1, although the updated model performed better during pH1N1, especially during the summer months.

## Introduction

In November, 2008, Google launched Google Flu Trends (GFT), an internet-based surveillance tool that uses aggregated Google search data to estimate influenza activity in near-real time [1]. To account for evolving online search behavior for health information, GFT models are updated annually using the most recent official surveillance data, where available, and to utilize any newly developed modeling techniques. In the United States, GFT models outpatient influenza-like illness (ILI) using publicly available ILI surveillance data provided by the U.S. Centers for Disease Control and Prevention (CDC). The CDC's sentinel provider surveillance system, known as ILINet, is a collaborative effort between the CDC, state and local health departments, and health care providers that estimates weekly the proportion of health care provider visits that are due to ILI [2].

Estimates of ILI produced by the GFT model developed in 2008 correlated highly with historical CDC ILI data [3], and GFT has since expanded to include 28 countries and 39 languages [1]. In Australia, a comparison of GFT with prospectively collected sentinel surveillance data collected from two systems showed ‘remarkable’ correlation between the systems [4]. A similar evaluation in New Zealand found that patterns from GFT were congruent with national surveillance systems, though inconsistencies were identified during pH1N1 circulation [5].

During the five years of data on which the original GFT model for the United States was built and tested, only seasonal influenza outbreaks occurred [6]. Previous commentary on the utility of GFT has expressed concern that it may be limited by the consistency of online health-seeking behavior [7]. Such a shift in behavior could occur during an outbreak or pandemic, resulting in a change in the terminology used to search online for health information. Thus, an open question was whether GFT could provide accurate estimates of non-seasonal flu. Should a new flu virus emerge and cause the same symptoms as seasonal flu, we expected GFT to detect it as long as Google users continued searching for similar flu-related terms; however, in the absence of non-seasonal flu outbreaks, there was no way to test this hypothesis.

In the spring of 2009 a new strain of influenza, pandemic influenza A (H1N1) [pH1N1], emerged, beginning in Mexico and quickly spreading to the United States and around the world [8]. The original U.S. GFT model was used to produce prospective estimates of ILI activity for the 2008–2009 flu season and retrospective estimates from 2003–2008. The updated model launched on September 24, 2009 incorporated ILINet data from April–September, 2009 and was used to produce both prospective estimates of ILI from September–December 2009 and retrospective estimates from July 2003 through September 2009. We compare the two models' composition and performance throughout 2009 and discuss changes in aggregated search counts on Google during the introduction of pH1N1 in the U.S.

## Materials and Methods

### Google Flu Trends Model

The derivation of the original GFT model has been described previously [3]. Briefly, we used aggregated search query data to estimate influenza activity in near-real time. We built a database that included time series of weekly counts for 50 million of the most common search queries in the United States. A query was defined as a complete exact sequence of terms issued by a Google search user. Separate aggregate weekly counts were kept for every query in each state. No information about the identity of any user was retained. A set of influenza-related queries was chosen using a sequential correlation-based method, and the proportion of outpatient visits that are ILI-related was estimated from the proportion of Google queries that are influenza-related using a linear model on the log-odds scale [3]. One season of influenza data was held out during model-fitting and then used to test the model estimated from the other seasons' data. The correlations between ILINet and GFT estimates for the held-out season were comparable to the correlations for the seasons used in model-fitting, which suggested that we were not over-fitting.

To update the model, the same basic methodology was employed. Surveillance data for ILI included the same time frame used in the original model (September 28, 2003 to May 11, 2008) and the first several months of the pH1N1 pandemic (March 29, 2009 through September 13, 2009). The updated model also selected from a larger candidate pool of queries since less common queries were allowed.

### Comparing the Original and Updated Models

We examined the composition of the original and updated models by comparing the number of selected queries and query volume (i.e., total number of searches for each query) in each, as well as by grouping the model queries into topic categories and comparing the relative query category volume (Table 1). A query category's relative volume was the combined volume of all queries in that category divided by the combined volume of all model queries.

**Table 1:** Comparison of relative query category volume in original and updated United States GFT models.

Query Category	Sample Query	Original Model Relative Category Volume	Updated Model Relative Category Volume
Symptoms of an influenza complication	[symptoms of bronchitis]	6%	11%
Influenza complication	[pnumonia]*	42%	6%
Specific influenza symptom	[fever]	6%	39%
General influenza symptoms	[early signs of the flu]	2%	30%
Cold/flu remedy	[robitussin]	12%	4%
Term for influenza	[influenza a]	<1%	3%
Antibiotic medication	[amoxicillin]	12%	0%
Related disease	[strep throat]	16%	<1%

*Search users often misspell the word *pneumonia*.

**Figure 1:**
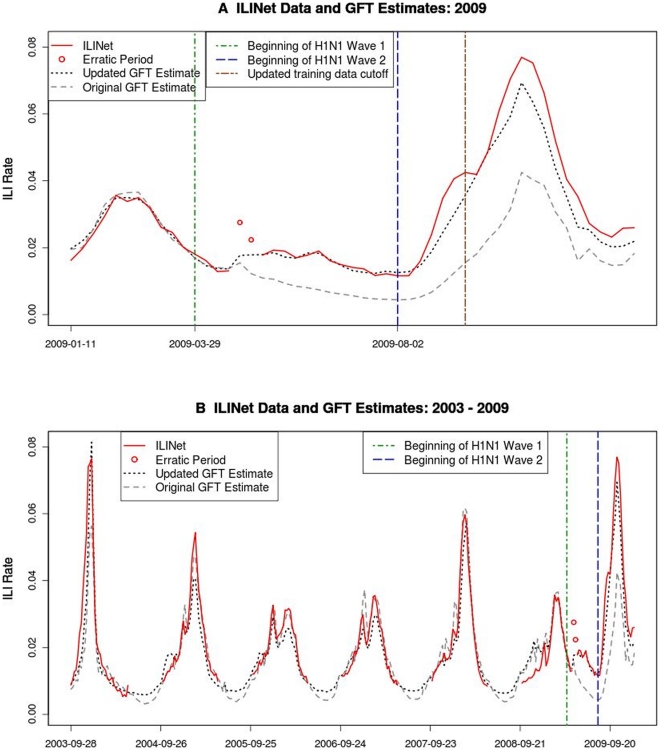
Time series plots of ILINet data and original and updated GFT estimates. A) ILINet data and GFT estimates from 2009. B) ILINet data and GFT estimates for the entire time period where GFT estimates are available: 2003–2009.

We compared the performance of the two models by calculating the Pearson correlation between model estimates and ILINet data. Since correlations largely measure consistency in the temporal alignment of the two time series, we also calculated the root mean square error (RMSE) to measure differences in the magnitude of the ILI and GFT estimates. We defined the following time periods for comparison: “pre-pH1N1”, defined as September 28, 2003 through March 29, 2009, and “pH1N1 period” defined as March 29, 2009 through December 31, 2009. The pH1N1 period was further divided into “pH1N1 Wave 1”, defined as March 29, 2009 through August 2, 2009, and “pH1N1 Wave 2”, defined as August 2, 2009 through December 31, 2009. The cutoff between the two waves corresponds approximately to when ILI rates began increasing towards the peak seen in October, 2009 [9]. The weeks of April 27, 2009 and May 3, 2009 were excluded from the correlation and RMSE calculation, due to tremendous media attention during those weeks. Those two weeks are labeled as the “erratic period” in Figures 1–3.

**Figure 2 pone-0023610-g002:**
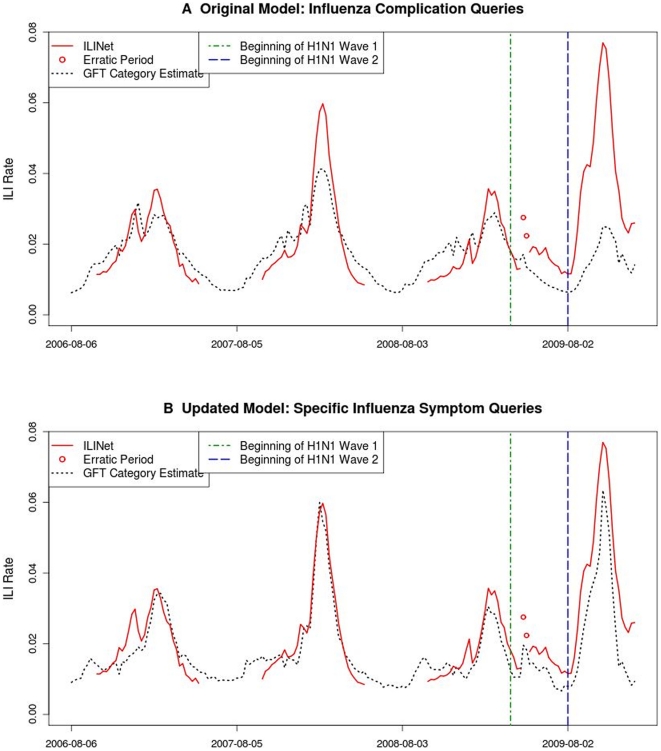
Time series plots of ILINet data and category-level GFT estimates. Category-level estimates are created by applying the GFT methodology to a subset of the queries in a given model. A) ILINet data and GFT estimates based on original model queries related to influenza complications. B) ILINet data and GFT estimates based on updated model queries related to specific influenza symptoms.

**Figure 3:**
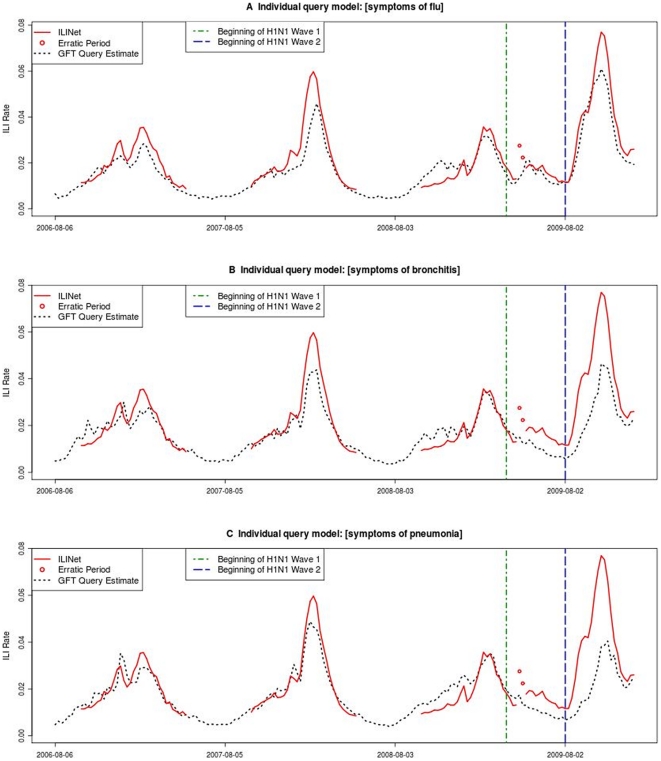
Time series plots of ILINet data and query-level GFT estimates. Query-level estimates are created by applying the GFT methodology to the search activity for a single query. A) ILINet data and GFT estimates based on the query [symptoms of flu]. B) ILINet data and GFT estimates based on the query [symptoms of bronchitis]. C) ILINet data and GFT estimates based on the query [symptoms of pneumonia].

To evaluate changes in search behavior during pH1N1, we examined query volume within and between query categories. We compared category volume during pH1N1 Wave 1 and Wave 2 with pre-pH1N1 volume. In addition, we developed several GFT models based on individual categories or queries to examine category- and query-level search trends. Graphical representations of the time series of the resulting model estimates and ILINet data were created to show how the relationship between a query category or individual query and ILI rates changed during pH1N1.

The original and updated Flu Trends models were built using C++ in a distributed computing framework [10]. All other analyses were performed using R [11], an open-source programming language for statistical analysis.

## Results

During the pre-pH1N1 and the pH1N1 period overall, both models' estimates were highly correlated with ILINet data (Table 2). However, during pH1N1 Wave 1, the original model did not correlate highly with ILINet data (0.290), whereas the updated model showed high correlation with ILINet data (r = 0.945). Both the original and updated model estimates were highly correlated with ILINet data during pH1N1 Wave 2 (r = 0.916 and r = 0.985 respectively).

**Table 2:** Correlation and RMSE between United States Google Flu Trends estimates and ILINet data.

	Pre-pH1N1(September 2003–March 2009)	pH1N1 Overall(March 2009–December 2009)	pH1N1 Wave 1(March 2009–August 2009)	pH1N1 Wave 2(August 2009–December 2009)
Correlation				
Original Model	0.906	0.912	0.290*	0.916
Updated Model	0.942	0.989	0.945	0.985
RMSE				
Original Model	0.006	0.018	0.008	0.023
Updated Model	0.005	0.005	0.001	0.007

*The overall correlation during pH1N1 is not an average of the Waves 1 and 2 correlations. The range of ILI rates was larger in Wave 2 than in Wave 1, causing the Wave 2 data to contribute more than the Wave 1 data to the overall correlation during pH1N1.

The magnitude of the ILI activity estimated by the original model was lower than both ILINet and the updated model estimates during the pH1N1 period overall, as evidenced by the threefold increase in RMSE compared to the pre-pH1N1 period (Table 2). The overall data trend for the two models was comparable, however, as evidenced by both models' high (r>0.9) correlations with ILINet data. The peak estimates of ILI activity occur during the same week in both models, and coincide with peak ILI activity as measured by ILINet (Figure 1).

We can also see from Figure 1 that both models provided accurate estimates of ILINet data during early 2009, when seasonal influenza was circulating. Over the entire pre-pH1N1 period, the updated model slightly outperformed the original model, both in terms of correlation with ILINet data (original model: r = 0.906; updated model: r = 0.942) and RMSE (original model: RMSE = 0.006; updated model: RMSE = 0.005; see Table 2). The updated model's peaks coincided with ILINet in four of the six pre-pH1N1 seasons (2003–04, 2004–05, 2005–06, and 2006–07); the original model's peaks coincided with ILINet in three previous seasons (2003–04, 2005–06, and 2007–08).

### Model Composition

The updated model included approximately 160 search query terms related to influenza activity, compared with approximately 40 in the original model. Although the updated model uses four times as many queries as the original model, it has only one-fourth the query volume of the original model due to the inclusion of less common queries than in the original model. The two models share 11 queries, which comprise 50% of the updated model's query volume but only 11% of the original model's query volume.

The updated model queries are more directly related to influenza, rather than complications associated with influenza infection, such as “pnumonia” (misspelling is intentional and reflects the actual query spelling), which were a large composition of the original model (Table 1). Queries in the categories “influenza complication” and “symptoms of an influenza complication” made up 48% of the volume of the original model; in the updated model, these categories comprise only 17% of the volume. Queries in the categories “general influenza symptoms” and “specific influenza symptoms” comprise 69% of the updated model volume, compared with only 8% of original model volume. In addition, 72% of the updated model queries contain the word ‘flu’ (38% of volume), compared to only 14% of original model queries (2% of volume).

### Search Behavior During pH1N1

Throughout the pH1N1 period, the total query volume for queries in the original model was lower than expected, given the previous relationship with ILINet data, and the original model therefore underestimated ILI activity. During the pH1N1 period, the original model underestimated ILINet data by an average of 0.014, a near three-fold increase in average error compared to the next-least-accurate season (2003), and a more than five-fold increase relative to the six prior seasons overall. Search query volume was low for nearly all query categories. In single-category models created to examine the volume decrease, all but one query category produced underestimates during the pH1N1 period. For example, queries in the category “influenza complication,” which previously comprised >40% of the original model query volume, underestimated ILINet data throughout the pH1N1 period (Figure 2). Queries in the category “term for influenza” had elevated volume during the early months of the pH1N1 period; however, these queries comprised a small portion of the model volume (approximately 1%). Similarly, an additional analysis of regional-level models showed that the original model underestimated ILINet data in all ten U.S. regions as well as nationally (data not shown).

Figure 3 shows ILINet data and estimates from single-query models for the original-model queries [symptoms of flu], [symptoms of bronchitis], and [symptoms of pneumonia]. Prior to pH1N1, all three queries closely tracked ILINet data. During the pH1N1 pandemic, [symptoms of flu] continued to closely track ILINet data, whereas [symptoms of bronchitis] and [symptoms of pneumonia] clearly underestimated ILINet data, especially during pH1N1 Wave 2.

The relative volume of several updated model query categories changed during the pH1N1 period (Table 3); still, the overall model volume accurately estimated ILINet data throughout the pH1N1 period. During pH1N1 Wave 1, the relative volume for the “specific influenza symptom” category decreased by 28% (Figure 2), and the relative volume for the “term for influenza” category increased by a factor of 2.5. During pH1N1 Wave 2, compared to pH1N1 Wave 1, the relative volume for the category “specific influenza symptom” decreased by a further 28%, and the relative volume for the category “general influenza symptoms” increased by 35%.

**Table 3:** Category-level query volume before and during the pH1N1 pandemic in the updated United States GFT model.

Query Category	Pre-pH1N1 Relative Category Volume	Wave 1 pH1N1 Relative Category Volume	Wave 2 pH1N1 Relative Category Volume
Specific influenza symptom	39%	28%	20%
General influenza symptoms	30%	28%	38%
Term for influenza	3%	11%	8%
Symptoms of an influenza complication	11%	15%	15%
Influenza complication	6%	6%	4%
Related disease	<1%	<1%	<1%
Cold/flu remedy	4%	3%	4%

## Discussion

The pH1N1 pandemic of 2009 provided the first opportunity to evaluate the performance of GFT models during a non-seasonal influenza outbreak. In September, 2009, Google implemented a planned annual update to its GFT model for the United States, and we were therefore able to evaluate the performance of two different models both prior to and during the pH1N1 epidemic.

Our analysis compared the two models and evaluated their correlation with ILINet data. The original model estimates were highly correlated with ILINet data during the pH1N1 period overall and Wave 2 specifically, but did not maintain this correlation during pH1N1 Wave 1, which was the introduction period for the virus into the United States. The original model's performance during pH1N1 illustrates that a high correlation across one time period (e.g., an influenza season) does not necessarily imply high correlation during smaller intervals (e.g., the initial acceleration of ILI rates). The updated model, which included the pH1N1 Wave 1 period in the training period, produced estimates that were highly correlated with ILINet data during pH1N1 overall and during Wave 1 and Wave 2 specifically. Both models' estimates peaked during the same week as ILINet. Both models' estimates were highly correlated with ILINet data prior to the pH1N1 pandemic, with correlation slightly higher for the updated model than for the original model.

While it is difficult to determine what precisely caused the change in flu-related search behavior, there are several possible explanations for why the original GFT model underestimated influenza activity during the pH1N1 pandemic. Firstly, users were searching less for queries related to influenza complications such as bronchitis and pneumonia (Figure 3), and this category comprised a large portion of the original model's query volume. Secondly, the pH1N1 virus emerged during the spring and summer months, rather than the fall and winter months typical for seasonal influenza. People may search using different query terms when ill with flu in the winter versus the summer. Finally, the CDC ILINet surveillance data, on which GFT data are trained, are based on reports from a variety of healthcare provider types, and may differ from true ILI rates [2]. Because ILINet estimates the proportion of outpatient visits that are due to ILI, ILINet data depend on both the underlying rate of influenza and also on the proportion of people with ILI symptoms seeking health care. A change in the latter could lead to a divergence between Flu Trends estimates and ILINet data. In particular, there is some evidence that during pH1N1 Wave 1, the proportion of outpatient visits due to ILI captured in ILINet was slightly elevated (61%) compared with Wave 2 (43%), due to ill persons more readily seeking health care for relatively mild illness during the first weeks of pH1N1 [12], [13]. Queries such as “swine flu” were popular during the pH1N1 pandemic and likely accounted for some of the changes in search behavior; however, such pandemic-specific queries are not included in GFT models because they do not correlate well with ILINet data in previous seasons, nor are they necessarily expected to correlate with future seasonal or non-seasonal influenza activity.

Google Flu Trends can provide timely and accurate estimates of the influenza activity in the United States, especially during peak activity, even in the wake of a novel form of influenza. Although more experience is needed to fully understand GFT performance during smaller waves and off-peak periods, the pH1N1 pandemic allowed us to build a GFT model incorporating both seasonal and pandemic influenza, which gives us added confidence in the ability of GFT to accurately estimate future influenza activity. Validation with surveillance systems monitoring laboratory-confirmed influenza disease are needed with nonspecific systems such as the one described here. Two GFT United States models were compared during the pH1N1 pandemic: an original model trained without pH1N1 data and an updated model trained on data including the initial wave of pH1N1, the summer months of the pandemic. The two models performed well prior to pH1N1 (r>0.9), with the updated model performing slightly better. Although the original model did not perform well during the initial wave of pH1N1, it did perform well during the second wave. Finally, the updated model accounted for the shift in search behavior and ILINet estimates, and performed well over both waves. We will continue to perform annual updates of Flu Trends models to account for additional changes in behavior, should they occur.
